# Lossy State Communication over Fading Multiple Access Channels

**DOI:** 10.3390/e25040588

**Published:** 2023-03-29

**Authors:** Viswanathan Ramachandran

**Affiliations:** KTH Royal Institute of Technology, 114 28 Stockholm, Sweden; visra@kth.se

**Keywords:** joint source-channel coding, joint compression and error correction, distortion-rate trade-off region, multiple access channels, fading channels, MMSE, dirty paper coding

## Abstract

Joint communications and sensing functionalities integrated into the same communication network have become increasingly relevant due to the large bandwidth requirements of next-generation wireless communication systems and the impending spectral shortage. While there exist system-level guidelines and waveform design specifications for such systems, an information-theoretic analysis of the absolute performance capabilities of joint sensing and communication systems that take into account practical limitations such as fading has not been addressed in the literature. Motivated by this, we undertake a network information-theoretic analysis of a typical joint communications and sensing system in this paper. Towards this end, we consider a state-dependent fading Gaussian multiple access channel (GMAC) setup with an additive state. The state process is assumed to be independent and identically distributed (i.i.d.) Gaussian, and non-causally available to all the transmitting nodes. The fading gains on the respective links are assumed to be stationary and ergodic and available only at the receiver. In this setting, with no knowledge of fading gains at the transmitters, we are interested in joint message communication and estimation of the state at the receiver to meet a target distortion in the mean-squared error sense. Our main contribution here is a complete characterization of the distortion-rate trade-off region between the communication rates and the state estimation distortion for a two-sender GMAC. Our results show that the optimal strategy is based on static power allocation and involves uncoded transmissions to amplify the state, along with the superposition of the digital message streams using appropriate Gaussian codebooks and dirty paper coding (DPC). This acts as a design directive for realistic systems using joint sensing and transmission in next-generation wireless standards and points to the relative benefits of uncoded communications and joint source-channel coding in such systems.

## 1. Introduction

The scarcity of spectrum, as well as the bandwidth requirements of key emerging applications such as 6G, necessitate a rethinking of resource consumption. In such systems, it appears prudent to co-design sensing and communication functionalities. This method enables significant gains in spectral, energy, hardware, and cost efficiency. This is known as joint sensing and communication, and it represents a paradigm shift in which sensing and communication operations can be jointly optimized by utilizing a single hardware platform and a joint signal processing framework. These ideas have already been used in a number of novel applications, including vehicular networks, indoor positioning, and covert communications. Joint sensing and communication scenarios have recently received a lot of attention from the signal processing community (see for instance [1,2,3,4]), the communications community (see [5,6,7,8,9,10,11]), and the information theory community (see [12,13,14,15,16]). This work belongs to the final category, where we take an information-theoretic view of joint sensing and communication in a multi-terminal setting.

**Figure 1:**
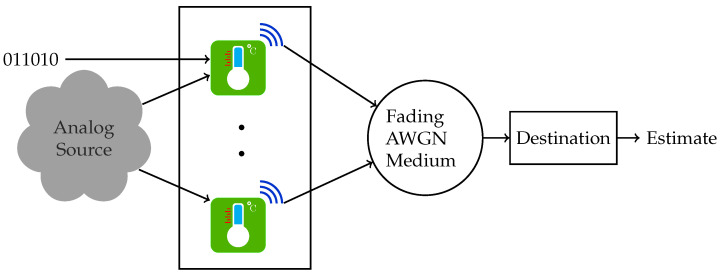
Sensor network example.

Joint sensing and communication also arises in multi-user networks with several sensor nodes observing a common analog source phenomenon, and communicating to a base station (destination) over a wireless fading medium, see Figure 1.

In this setting, the sensor nodes must convey a description of the source process to the base station, which then tries to estimate the source process subject to a fidelity criterion. Some of the sensor nodes might also have additional digital data to convey to the base station, which must reliably recover them as well. Since the source process, as well as the data from each node, are of interest to the base station, a tension naturally arises between the rates of data communication and source estimation fidelity. The trade-off between these objectives is of particular interest in such systems, which is among the primary motivations for this work. The analog phenomenon in this example can be thought of as a *channel state* that affects the digital communication of messages, with the receiver being required to reliably estimate this channel state while also recovering the transmitted messages. As far as the fading process is concerned, it is reasonable in practice to assume that the receiver can track the channel variations, for example, via the use of pilot transmission sequences.

In this work, we consider an information-theoretic abstraction of the communication setting in Figure 1. In particular, we focus on joint communication and state estimation over a state-dependent fading Gaussian multiple access channel with no fading knowledge at the transmitters. At each encoder, the state process is assumed to be known non-causally. The fading processes encountered on the respective links are assumed to be stationary and ergodic and to be known only at the receiver. The dual goals of message communication and state estimation at the receiver must be met with a distortion tolerance with respect to a squared-error metric. The trade-off between the average message communication rates and the average distortion in receiver state estimation is of interest. We completely characterize the optimal trade-off region between the communication rates of the different transmitters and the state estimation distortion at the receiver. The details of the setting as well as the motivation for investigating it, will be elucidated in the section that follows.

Having introduced the general problem framework, we now discuss the other relevant contributions in the literature, emphasizing the differences from our setting under consideration in the following section.

## 2. Literature Review

In this section, we discuss the related literature and place our contributions in the context of the state of the art. In particular, we enlist prior works on joint communication and channel state estimation in both point-to-point as well as network information theoretic settings and identify several knowledge gaps.

Systems such as the one in Figure 1 can be modeled as *state-dependent channels*, where the channel state typically refers to a variable used to model unknown parameters of the channel statistics. A canonical form of such state-dependent channel models consisting of an additive state over an additive white Gaussian noise (AWGN) channel was investigated in [17], popularly known as the *dirty paper coding* (DPC) setting. Surprisingly, ref. [17] demonstrated that regardless of the presence of the state, the channel capacity of this setting remained the same as that of an AWGN channel, independent of the variance of the channel state. This phenomenon later found widespread applications in settings such as digital watermarking [18] and multiple-input-multiple-output wireless broadcast channels [19].

In certain state-dependent channels, in addition to communicating messages, the transmitter may wish to assist the receiver in estimating the channel state (as in the sensor network scenario described in Figure 1). Splitting the average available power between the dual tasks of uncoded transmission of the state and DPC for the message was found to be optimal for the mean squared error distortion measure [20] in a point-to-point (single-user) AWGN channel. In [21], joint communication and state estimation were considered in a different scenario where the transmitters were unaware of the channel state. In an interesting variation of [20], Tian et al. [22] characterized the distortion-transmit power trade-off in a point-to-point Gaussian model with noisy state observations at the transmitter in the absence of messages. However, in the presence of messages [22], a complete characterization of the rate-distortion trade-off region remains unknown for the case of noisy state observations at the transmitter.

In the literature, channel state estimation has been studied in two different frameworks, each of them being motivated by different real-world problems. These frameworks correspond to (a) state estimation performed at the receiver and (b) state estimation performed at the transmitter side. We first consider the problem of channel state estimation at the receiver, which has been investigated before in certain information-theoretic settings. Relevant works include [23] (see also [24]), which investigated joint estimation and message communication over a Gaussian broadcast channel (BC) without state-dependence and derived a complete characterization of the trade-off between achievable rates and estimation errors. In [25], communication and state estimation were studied in a multiple access setting without fading, and the distortion-rate trade-off region was characterized. In [26], a state-dependent Gaussian BC with the dual goals of amplifying the channel state at one of the receivers while masking it [27] from the other receiver (with no message transmissions) was investigated, and achievable coding schemes, as well as outer bounds, were derived. More recently, ref. [28] addressed state estimation for a discrete memoryless BC with causal state information at the transmitter, also taking into account any possible feedback signals from the strong receiver to the sender, and gave a characterization of the capacity region.

As far as channel state estimation at the transmitter is concerned, this line of work originated in [12], where a point-to-point channel with generalized feedback signals to aid the state estimation was investigated. Such models are motivated by joint radar and communications systems where the radar, as well as data communication, share the same frequency band. Following this, ref. [13] considered a multiple access channel extension of the same, where both the senders obtained generalized feedback and obtained an achievable trade-off region. An improved achievable scheme for the multiple access setting was derived recently in [15]. Most recently, a broadcast channel variant was investigated in [16] (see also [14]), where inner and outer bounds were given for general broadcast channels while a complete characterization was obtained for the special case of physically degraded broadcast channels.

Fading multiple access channels without state (or state estimation requirements) and different degrees of channel state information have been explored in the literature. For instance, the ergodic capacity region for fast-fading Gaussian multiple access channels (GMAC) with perfect channel state information at the transmitters and the receiver was characterized in [29]. More general configurations for transmitter channel state availability were analyzed in [30], where the capacity region for time-varying models was determined via optimization over appropriate power control laws. Slow fading multiple access channels with distributed channel state information at the transmitters were studied in [31].

State-dependent point-to-point fading channels with no state estimation have received some attention in the literature. Vaze and Varanasi [32], for example, investigated a model with full state knowledge and partial knowledge of the fading process at the transmitter, and the high-SNR achievable rate was characterized. Rini and Shamai [33] examined the impact of phase fading in the DPC setting [17] when the receiver was informed of the fading process. We also note that [34] has addressed point-to-point fading channels (without any state process or state estimation requirements) with channel gains known at both the sender and the receiver.

Having reviewed the related literature, we now identify the key knowledge gaps in prior work and the necessity of our work.

Analysis of the State of the art and Research gaps: We identify the following crucial aspects.

- We note that none of the works above consider joint state estimation along with message communication over state-dependent multi-terminal settings with noncausal transmitter state information, which is highly relevant in applications like the joint sensing and communication setting in Figure 1. This is addressed in this paper.
- While there exist system-level guidelines and waveform design specifications for such systems, a network information-theoretic analysis of the absolute performance capabilities of joint sensing and communication systems that take into account practical limitations has not been addressed in the literature, which we undertake here.
- Moreover, none of the works on joint communication and estimation mentioned above take fading links into account. Fading is an impairment that must be accounted for in practical wireless communication channel models, such as the joint sensing and communication application shown in Figure 1. This is another gap in the literature that this paper seeks to fill by investigating joint communication and estimation over state-dependent multi-user fading channels, the point-to-point counterpart of which was addressed by the author in [35].

Novelty and relevance: In this paper, we address the problem of joint communication and state estimation over a state-dependent fading GMAC with no fading knowledge at the transmitters. The key scientific question we address here is: what is the best possible trade-off between the competing goals of message communication from multiple senders and the fidelity in state estimation at the receiver?

- The key novelty of our work is that it is the first instance where joint communication and estimation have been considered in a multiple-user setting that also accounts for fading links, as opposed to previous works, which focused only on non-fading links.
- Moreover, it is the first work that considers non-causal state information (as opposed to causal or strictly causal) at the transmitter in a fading multi-user scenario which is practically relevant as described in the sensor network example from Figure 1.
- Furthermore, we undertake a comprehensive network information-theoretic study of the fundamental performance limits of such joint communication and estimation settings, which is lacking in the literature. Please refer to Table 1, which highlights our contributions in this paper with respect to the existing works.

The key relevance of our study is that it serves as a design guideline for practical systems employing joint sensing and communication envisioned in future 6G wireless standards and broadly applies to systems that involve joint compression and communication/rate-distortion trade-offs. It also points to the relative benefits of uncoded transmission versus joint source-channel coding in such systems. The progress embodied herein builds up towards a better understanding of joint state estimation and communication problems in multi-terminal settings (such as multiple access channels), which is relatively less explored in the literature (with or without the fading aspect).

Summary of contributions: We list them below. See also the contribution summary Table 1, which emphasizes the novelty of our work with respect to the existing works.

- One of our main contributions in the paper is a complete characterization (Theorem 1) of the rate-distortion trade-off region for joint state estimation and communication over a two-user fading GMAC with the state. The key non-trivial part is the proof of converse, which is given in Section 5.
- We prove that the optimal strategy for the setting under consideration involves uncoded transmissions to amplify the state, along with the superposition of the digital message streams using appropriate Gaussian codebooks and DPC.
- We prove the optimality of uncoded state amplification in the special case where there are no messages to communicate—please refer to the section on implications of our result given after the statement of Theorem 1 for the details.
- Our framework naturally generalizes the results of [35] to multiple users, ref. [25] to fading links and the work of [20] to fading links with multiple users, thereby providing a unified framework that encompasses all these prior works on joint communication and estimation.
- Our study gives a network information-theoretic analysis of the fundamental performance limits of joint sensing and communication systems that take into account practical limitations such as fading. This acts as a design directive for realistic systems using joint sensing and transmission anticipated in upcoming wireless standards and points to the relative merits of uncoded communications and joint source-channel coding in such systems.

Notations: Random variables are denoted by capital letters, while their realizations are denoted by the corresponding lower-case letters. We use P(·) to denote the probability of an event. The joint probability distribution of two random variables (X,Y) is denoted by $p_{X,Y}\left(x,y\right)$. Let E[·] denote the expected value of a random variable. At times, we will use an explicit subscript in the expectation operation, $E_{X}\left[\cdot \right]$, to denote that the expectation is taken with respect to the probability distribution of the random variable *X*. All logarithms are in base 2, unless mentioned otherwise. We denote random sequences of length *n* with a superscript notation, i.e., $U^{n}:=U_{1},U_{2},\cdots ,U_{n}$. An indexed set of random sequences each of length *n* is denoted with a subscript for the random variable and a superscript for the length, i.e., $U_{j}^{n}:=U_{j1},U_{j2},\cdots ,U_{jn}$, where $U_{ji}$ stands for the i-th component of $U_{j}^{n}$. The covariance of a random vector $X^{n}$ is denoted by $\mathrm{Cov}(X^{n})$. Calligraphic letters represent alphabets of random variables. ∥.∥ denotes the Euclidean norm of a vector, while Conv(·) denotes the convex closure of a set. The absolute value of a number is denoted |·|, while the transpose of a matrix *A* is denoted as $A^{T}$. The notation A⫫B is used to denote independent random variables (A,B). The Gaussian (normal) distribution with mean μ and variance $\sigma^{2}$ is denoted by $N(\mu ,\sigma^{2})$. The set of real numbers is denoted by R, while the set of n−tuples of positive real numbers is denoted by $R_{+}^{n}$. The Shannon entropy of a discrete-valued random variable *X* is denoted by H(X), while the differential entropy of a continuous-valued random variable *Y* is denoted by h(Y). The mutual information between any two random variables *V* and *W* is denoted by I(V;W). The corresponding conditional quantities given a random variable *Z* are conditional entropy H(X|Z), conditional differential entropy h(Y|Z), and conditional mutual information I(V;W|Z). If *n* is an integer variable, ϕ(n) is a positive function and f(n) is an arbitrary function, we say that f=o(ϕ) provided that $\lim_{n\to \infty }f\left(n\right)/\phi \left(n\right)=0$. For any three random variables (A,B,C), we say that A→B→C is a Markov chain if *A* and *C* are conditionally independent given *B*.

The rest of the paper is organized as follows: in Section 3, we introduce the system model and state our main results. Section 4 and Section 5 contain the achievable coding scheme and the converse to the rate-distortion trade-off region, respectively. Section 6 contains concluding remarks. The Appendix A.1, Appendix A.2, and Appendix A.3 contain all the details of the proofs that are skipped in the main discussion, to maintain the readability of the paper.

## 3. System Model and Results

**Figure 2:**
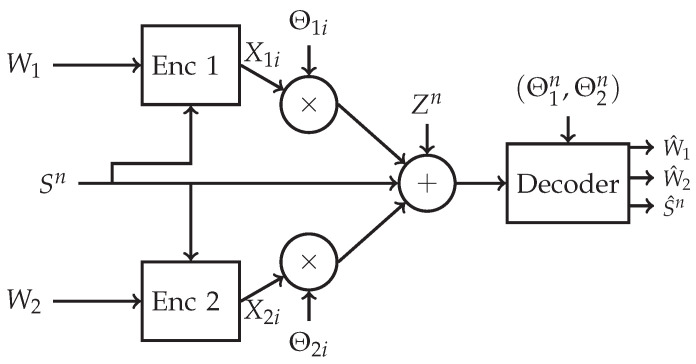
State estimation over a fading Gaussian MAC with state, without fading knowledge at the transmitters.

Consider the fading multiple access channel shown in Figure 2. The observed samples at the receiver at time instant i∈{1,2,…,n} are given by

(1) $$\begin{array}{c} Y_{i}=\sum_{j=1}^{2}\Theta_{j,i}X_{j,i}+S_{i}+Z_{i}. \end{array}$$

Here, the samples of the additive noise process are independent and identically distributed (i.i.d.) according to $Z_{i}∼N\left(0,\sigma_{Z}^{2}\right)$, while the samples of the state process are i.i.d. according to $S_{i}∼N\left(0,\sigma_{S}^{2}\right)$. The state process is assumed to be known non-causally at each encoder. The fading processes encountered on the respective links are represented by $\Theta_{j}^{n},j\in \left\{1,2\right\}$, with these fading gains being known only at the receiver. The fading processes encountered on both links are assumed to be stationary and ergodic. The codeword lengths can be chosen arbitrarily long to average over the fading of the channel. The given model represents a fast-fading multiple access channel with no knowledge of the fading processes at the transmitters. The state, fading, and additive noise processes are assumed to be independent of each other. In our model, the power constraint on the inputs is assumed to be across blocks, averaged over the random state and the codebook. The dual goals of message ($\left(W_{1},W_{2}\right)$ in Figure 2) communication, and estimation of the state $S^{n}$ at the receiver to meet a distortion tolerance with respect to a squared error metric must be met. The trade-off between the average message communication rates ($R_{1},R_{2})$ and the average distortion incurred in state estimation (*D*) at the receiver is sought.

We take $W_{j}$ to be uniformly drawn from the set $W_{j}≜\left\{1,2,\cdots ,2^{nR_{j}}\right\}$ for j={1,2}, and independent of each other. The messages $\left(W_{1},W_{2}\right)$ are assumed to be independent of the state process $S^{n}$. The power constraint on the transmissions is:

(2) $$\frac{1}{n}E_{W_{j},S^{n}}\left[\sum_{i=1}^{n}X_{ji}^{2}\left(W_{j},S^{n}\right)\right]\leq P_{j},\;\;j=\left\{1,2\right\}.$$

After *n* observations, the decoder estimates ${\hat{S}}^{n}=\phi \left(Y^{n},\Theta_{1}^{n},\Theta_{2}^{n}\right)$ using a (state) reconstruction map $\phi \left(\cdot \right):Y^{n}\times \prod_{j=1}^{2}\Theta_{j}^{n}\to R^{n}$, and also decodes the messages $\left(W_{1},W_{2}\right)$. The (message) decoding map is given by $\psi :Y^{n}\times \prod_{j=1}^{2}\Theta_{j}^{n}\to W_{1}\times W_{2}$. Our aim here is to maintain the average squared-error distortion incurred in state estimation below a given threshold, while also ensuring that the average error probability of decoding the messages is small enough.

**Definition 1.**  *A scheme using the encoder mappings $E_{j}:\left\{1,\cdots ,2^{nR_{j}}\right\}\times S^{n}\to X_{j}$ for j=1,2 satisfying the power constraints in (2), along with two mappings ϕ(·) and ψ(·) at the receiver is called an $\left(n,R_{1},R_{2},P_{1},P_{2}\right)$ communication-estimation scheme.*

A triple $\left(R_{1},R_{2},D\right)$ is said to be achievable if there exists a sequence of $\left(n,R_{1},R_{2},P_{1},P_{2}\right)$ communication-estimation schemes such that

(3) $$\begin{array}{cc} \underset{n\to \infty }{lim sup}\frac{1}{n}E_{\Theta_{1}^{n},\Theta_{2}^{n}}\left\{E\left[∥S^{n}-\phi \left(Y^{n},\Theta_{1}^{n},\Theta_{2}^{n}\right){∥}^{2}\right]\right\} & \leq D, \end{array}$$

and

(4) $$\begin{array}{cc} \underset{n\to \infty }{lim sup}E_{\Theta_{1}^{n},\Theta_{2}^{n}}\left\{P(\psi \left(Y^{n},\Theta_{1}^{n},\Theta_{2}^{n}\right)\neq \left(W_{1},W_{2}\right))\right\} & =0. \end{array}$$

Let $C_{\mathrm{est}}^{\mathrm{fad}-\mathrm{mac}}\left(P_{1},P_{2}\right)$ be the closure of the set of all achievable $\left(R_{1},R_{2},D\right)$ triples, with $0\leq D\leq \sigma_{S}^{2}$. The main result of the paper is stated below.

**Theorem 1.**  *For the fading Gaussian MAC with state, the trade-off region $C_{\mathrm{est}}^{\mathrm{fad}-\mathrm{mac}}\left(P_{1},P_{2}\right)$ is characterized by the convex closure of all $\left(R_{1},R_{2},D\right)\in R_{+}^{3}$ such that*

(5) $$\begin{array}{c} R_{1}\leq E_{\Theta_{1}}\left[\frac{1}{2}\log\left(1+\frac{\gamma \Theta_{1}^{2}P_{1}}{\sigma_{Z}^{2}}\right)\right], \end{array}$$

(6) $$\begin{array}{c} R_{2}\leq E_{\Theta_{2}}\left[\frac{1}{2}\log\left(1+\frac{\beta \Theta_{2}^{2}P_{2}}{\sigma_{Z}^{2}}\right)\right], \end{array}$$

(7) $$\begin{array}{c} R_{1}+R_{2}\leq E_{\Theta_{1},\Theta_{2}}\left[\;\frac{1}{2}\;\log\left(\;1+\;\frac{\gamma \Theta_{1}^{2}P_{1}\;+\;\beta \Theta_{2}^{2}P_{2}}{\sigma_{Z}^{2}}\;\right)\;\right]\;, \end{array}$$

(8) $$\begin{array}{c} D\geq E_{\Theta_{1},\Theta_{2}}\left[\;\frac{\sigma_{S}^{2}\left(\sigma_{Z}^{2}\;+\;\gamma \Theta_{1}^{2}P_{1}\;+\;\beta \Theta_{2}^{2}P_{2}\right)}{\left(\begin{array}{cc} & \Theta_{1}^{2}P_{1}\;+\;\Theta_{2}^{2}P_{2}\;+\;\sigma_{S}^{2}\;+\;\sigma_{Z}^{2}\;+\;2\Theta_{1}\sqrt{\bar{\gamma }P_{1}\sigma_{S}^{2}}\;\\ & +\;2\Theta_{2}\sqrt{\bar{\beta }P_{2}\sigma_{S}^{2}}\;+\;2\Theta_{1}\Theta_{2}\sqrt{\bar{\gamma }\bar{\beta }P_{1}P_{2}} \end{array}\right)}\;\right]\;, \end{array}$$

*for some fractions γ∈[0,1] and β∈[0,1], with $\bar{\gamma }≜1-\gamma$ and $\bar{\beta }≜1-\beta$.*

**Proof.**  The proof is given in the following two sections, wherein Section 4 contains the achievability proof while the converse is proved in Section 5. □

Implications of our result: We now discuss the main consequences of our Theorem 1 for the sensor network scenario outlined earlier in Figure 1. If a given transmitter (sensor node) has a message to communicate to the receiver (base station), then the optimal strategy involves splitting its available power budget into two parts: one part is used to send a scaled version of the state (uncoded state amplification), while the other part is used to communicate the message using dirty paper coding. The parameters γ and β in Theorem 1 precisely perform this role of power-sharing between the dual goals of communication and estimation. This will be elaborated upon in the proof of achievability in Section 4.

On the other hand, if a given transmitter (sensor node) has no messages to communicate to the receiver (base station), then the optimal strategy simply involves utilizing its entire power budget for uncoded state amplification, i.e., sending the scaled version

(9) $$\begin{array}{c} X_{j}=\sqrt{\frac{P_{j}}{\sigma_{S}^{2}}}S. \end{array}$$

In other words, uncoded transmission is optimal for such nodes. This phenomenon resembles that of [38], albeit the latter is in the context of non-fading links with no message communication and no state-dependence. We close this section with a series of remarks that shed further light on the implications of our Theorem 1 and its connection with existing results in the literature.

**Remark 1.**  *When the second sender is absent, i.e., $P_{2}=0$, and with constant fading gains $\Theta_{1}\left(=\Theta_{2}\right)=1$ almost surely, our Theorem 1 recovers the characterization of [20] for the point-to-point non-fading scenario as a special case.*

**Remark 2.**  *When the fading gains are constant, i.e., $\Theta_{1}=\Theta_{2}=1$ almost surely, our Theorem 1 recovers the characterization of [25] for the multiple-access non-fading scenario as a special case.*

**Remark 3.**  *When γ=β=0, we obtain an extreme point of the region with zero rates, i.e, $R_{1}=R_{2}=0$, and the best state estimate, i.e., minimum possible distortion*

(10) $$\begin{array}{c} D_{\min}=E_{\Theta_{1},\Theta_{2}}\left[\;\frac{\sigma_{S}^{2}\sigma_{Z}^{2}}{\left(\begin{array}{cc} \; & \Theta_{1}^{2}P_{1}\;+\;\Theta_{2}^{2}P_{2}\;+\;\sigma_{S}^{2}\;+\;\sigma_{Z}^{2}\;+\;2\Theta_{1}\sqrt{P_{1}\sigma_{S}^{2}}\;\\ & +\;2\Theta_{2}\sqrt{P_{2}\sigma_{S}^{2}}\;+\;2\Theta_{1}\Theta_{2}\sqrt{P_{1}P_{2}} \end{array}\right)}\;\right]. \end{array}$$

*This corresponds to each encoder utilizing its entire power budget for uncoded state amplification, and therefore no message communication is possible.*

**Remark 4.**  *On the other hand, when γ=β=1, we obtain the other extreme point of the region with the maximum possible rates for a fading Gaussian MAC, and the worst state estimate, i.e., maximum possible distortion*

(11) $$\begin{array}{c} R_{1}\leq R_{1,\max}=E_{\Theta_{1}}\left[\frac{1}{2}\log\left(1+\frac{\Theta_{1}^{2}P_{1}}{\sigma_{Z}^{2}}\right)\right], \end{array}$$

(12) $$\begin{array}{c} R_{2}\leq R_{2,\max}=E_{\Theta_{2}}\left[\frac{1}{2}\log\left(1+\frac{\Theta_{2}^{2}P_{2}}{\sigma_{Z}^{2}}\right)\right], \end{array}$$

(13) $$\begin{array}{c} R_{1}+R_{2}\leq E_{\Theta_{1},\Theta_{2}}\left[\;\frac{1}{2}\;\log\left(\;1+\;\frac{\Theta_{1}^{2}P_{1}\;+\;\Theta_{2}^{2}P_{2}}{\sigma_{Z}^{2}}\;\right)\;\right]\;, \end{array}$$

(14) $$\begin{array}{c} D\geq D_{\max}=E_{\Theta_{1},\Theta_{2}}\left[\;\frac{\sigma_{S}^{2}\left(\sigma_{Z}^{2}\;+\;\Theta_{1}^{2}P_{1}\;+\;\Theta_{2}^{2}P_{2}\right)}{\left(\Theta_{1}^{2}P_{1}\;+\;\Theta_{2}^{2}P_{2}\;+\;\sigma_{S}^{2}\;+\;\sigma_{Z}^{2}\right)}\;\right]\;, \end{array}$$

*This corresponds to each encoder utilizing its entire power budget for message communication, and therefore no state amplification is possible, and maximum distortion is incurred in state estimation.*

## 4. Achievability

The achievability builds upon well-known techniques like dirty paper coding and successive cancellation, along with appropriate power splitting. The power $P_{1}$ available at encoder 1 is split into two parts: $\gamma P_{1}$ for message transmission and $\bar{\gamma }P_{1}$ for state amplification, for some γ∈[0,1]. Similarly, the power $P_{2}$ available at the second encoder is split into $\beta P_{2}$ (message transmission) and $\bar{\beta }P_{2}$ (state amplification) for some β∈[0,1]. Then, the following state amplification signals are generated

(15) $$X_{1sj}=\sqrt{\frac{\bar{\gamma }P_{1}}{\sigma_{S}^{2}}}S_{j}\;\;\mathrm{and}\;\;X_{2sj}=\sqrt{\frac{\bar{\beta }P_{2}}{\sigma_{S}^{2}}}S_{j},\;1\leq j\leq n$$

at the respective encoders. In other words, the power fractions $\bar{\gamma }P_{1}$ and $\bar{\beta }P_{2}$ at encoders 1 and 2 respectively are used for uncoded state amplification. Hence, (1) can be rewritten as

(16) Yj=Θ1jX1mj+Θ1jX1sj+Θ2jX2mjwww+Θ2jX2sj+Sj+Zj=Θ1jX1mj+Θ2jX2mjwww+1+Θ1jγ¯P1σS2+Θ2jβ¯P2σS2Sj+Zj,

where $E[||X_{1m}^{n}{\left|\right|}^{2}]\leq n\gamma P_{1}$ and $E[||X_{2m}^{n}{\left|\right|}^{2}]\leq n\beta P_{2}$, with both $X_{1m}^{n}$ and $X_{2m}^{n}$ being independent of $S^{n}$. The subscript m in (16) indicates that the corresponding signals are intended for message transmission, whereas the subscript s indicates state amplification signals. To communicate the messages across to the receiver, we invoke the writing on dirty paper result for a Gaussian MAC [37].

From the DPC result [17], we recall that a known state process over an AWGN channel can be completely canceled. In particular, a rate *R* that satisfies

(17) R≤I(U;Y)−I(U;S),

when evaluated for some feasible joint probability distribution $p_{U,S,X}\left(u,s,x\right)p_{Y|X,S}\left(y|x,s\right)$, can be achieved by Gelfand-Pinsker coding [39] for a point-to-point channel with a non-causally known state. In order to prove the achievability of the rates (5)–(7), we first consider a dirty paper channel with input $\Theta_{1j}X_{1mj}$, known state

$$\begin{array}{c} S_{j}^{\prime }=\left(1+\Theta_{1j}\sqrt{\bar{\gamma }P_{1}/\sigma_{S}^{2}}+\Theta_{2j}\sqrt{\bar{\beta }P_{2}/\sigma_{S}^{2}}\right)S_{j}, \end{array}$$

and unknown noise $\Theta_{2j}X_{2mj}+Z_{j}$. We choose $U_{1j}=\Theta_{1j}X_{1mj}+\alpha_{1j}S_{j}^{\prime }$, $X_{1mj}⫫S_{j}$ with $X_{1mj}∼N\left(0,\gamma P_{1}\right)$ and

$$\begin{array}{c} \alpha_{1j}=\frac{\gamma \Theta_{1j}^{2}P_{1}}{\gamma \Theta_{1j}^{2}P_{1}+\beta \Theta_{2j}^{2}P_{2}+\sigma_{Z}^{2}}. \end{array}$$

This yields the following rate for user-1 at time instant *j* with the error probability approaching zero

(18) $$\begin{array}{cc} \; & \frac{1}{2}\log\left(1+\frac{\gamma \Theta_{1j}^{2}P_{1}}{\beta \Theta_{2j}^{2}P_{2}+\sigma_{Z}^{2}}\right). \end{array}$$

The achievable rate for user-1 averaged over a time interval {1,2,…,n} is

(19) $$\begin{array}{cc} \; & \frac{1}{n}\sum_{j=1}^{n}\frac{1}{2}\log\left(1+\frac{\gamma \Theta_{1j}^{2}P_{1}}{\beta \Theta_{2j}^{2}P_{2}+\sigma_{Z}^{2}}\right), \end{array}$$

which converges as n→∞ to

(20) $$\begin{array}{cc} \; & E\left[\frac{1}{2}\log\left(1+\frac{\gamma \Theta_{1}^{2}P_{1}}{\beta \Theta_{2}^{2}P_{2}+\sigma_{Z}^{2}}\right)\right] \end{array}$$

due to the stationarity and ergodicity of the fading processes. The decoded codeword $U_{1j}$ is then subtracted from the channel output to obtain another dirty paper channel

$$\begin{array}{c} {\tilde{Y}}_{j}=Y_{j}-U_{1j}=\Theta_{2j}X_{2mj}+\left(1-\alpha_{1j}\right)S_{j}^{\prime }+Z_{j}, \end{array}$$

with input $\Theta_{2j}X_{2mj}$, known state $S_{j}^{\prime \prime }=\left(1-\alpha_{1j}\right)S_{j}^{\prime }$ and unknown noise $Z_{j}$. We pick $U_{2j}=\Theta_{2j}X_{2mj}+\alpha_{2j}S_{j}^{\prime \prime }$, $X_{2mj}⫫S_{j}$ with $X_{2mj}∼N\left(0,\beta P_{2}\right)$ and

$$\begin{array}{c} \alpha_{2j}=\frac{\beta \Theta_{2j}^{2}P_{2}}{\beta \Theta_{2j}^{2}P_{2}+\sigma_{Z}^{2}}. \end{array}$$

This yields the following rate for user-2 at time instant *j* with the error probability approaching zero

(21) $$\begin{array}{cc} \; & \frac{1}{2}\log\left(1+\frac{\beta \Theta_{2j}^{2}P_{2}}{\sigma_{Z}^{2}}\right). \end{array}$$

The achievable rate for user-2 averaged over a time interval {1,2,…,n} is

(22) $$\begin{array}{cc} \; & \frac{1}{n}\sum_{j=1}^{n}\frac{1}{2}\log\left(1+\frac{\beta \Theta_{2j}^{2}P_{2}}{\sigma_{Z}^{2}}\right), \end{array}$$

which converges as n→∞ to

(23) $$\begin{array}{cc} \; & E\left[\frac{1}{2}\log\left(1+\frac{\beta \Theta_{2}^{2}P_{2}}{\sigma_{Z}^{2}}\right)\right] \end{array}$$

due to the stationarity and ergodicity of the fading processes. By reversing the decoding order and using time-sharing, the region in expressions (5) through (7) can be achieved. Note that the right-hand sides of expressions (20) and (23) add up to the right-hand side of the sum rate expression in (7). For the state estimate, the receiver forms the linear minimum mean-squared error (MMSE) estimate ${\hat{S}}_{j}\left(Y_{j}\right)$ of $S_{j}$ based on $Y_{j}$:

$$\begin{array}{cc} {\hat{S}}_{j}\left(Y_{j}\right) & =\frac{\left(\sigma_{S}^{2}+\Theta_{1j}\sqrt{\bar{\gamma }P_{1}\sigma_{S}^{2}}+\Theta_{2j}\sqrt{\bar{\beta }P_{2}\sigma_{S}^{2}}\right)\;\;Y_{j}}{\left(\begin{array}{cc} & \Theta_{1j}^{2}P_{1}\;+\;\Theta_{2j}^{2}P_{2}\;+\;\sigma_{S}^{2}\;+\;\sigma_{Z}^{2}\;\\ & +\;2\Theta_{1j}\sqrt{\bar{\gamma }P_{1}\sigma_{S}^{2}}\;+\;2\Theta_{2j}\sqrt{\bar{\beta }P_{2}\sigma_{S}^{2}}\;\\ & +\;2\Theta_{1j}\Theta_{2j}\sqrt{\bar{\gamma }\bar{\beta }P_{1}P_{2}} \end{array}\right)}\\ \; & ≜\frac{c_{1}}{c_{2}}Y_{j}, \end{array}$$

where

$$\begin{array}{cc} c_{1} & =\sigma_{S}^{2}+\Theta_{1j}\sqrt{\bar{\gamma }P_{1}\sigma_{S}^{2}}+\Theta_{2j}\sqrt{\bar{\beta }P_{2}\sigma_{S}^{2}},\\ c_{2} & =\left(\begin{array}{cc} & \Theta_{1j}^{2}P_{1}\;+\;\Theta_{2j}^{2}P_{2}\;+\;\sigma_{S}^{2}\;+\;\sigma_{Z}^{2}\;\\ & +\;2\Theta_{1j}\sqrt{\bar{\gamma }P_{1}\sigma_{S}^{2}}\;+\;2\Theta_{2j}\sqrt{\bar{\beta }P_{2}\sigma_{S}^{2}}\;\\ & +\;2\Theta_{1j}\Theta_{2j}\sqrt{\bar{\gamma }\bar{\beta }P_{1}P_{2}} \end{array}\right). \end{array}$$

For the achievable distortion at time instant *j*, we evaluate the expected squared error between $S_{j}$ and ${\hat{S}}_{j}\left(Y_{j}\right)$, above. The resulting MMSE can be easily verified to be

$$\begin{array}{cc} E[|S_{j}-{\hat{S}}_{j}{|}^{2}] & =\sigma_{S}^{2}-\frac{c_{1}^{2}}{c_{2}}\\ \; & =\frac{\sigma_{S}^{2}\left(\sigma_{Z}^{2}\;+\;\gamma \Theta_{1j}^{2}P_{1}\;+\;\beta \Theta_{2j}^{2}P_{2}\right)}{\left(\begin{array}{cc} & \Theta_{1j}^{2}P_{1}\;+\;\Theta_{2j}^{2}P_{2}\;+\;\sigma_{S}^{2}\;+\;\sigma_{Z}^{2}\;\\ & +\;2\Theta_{1j}\sqrt{\bar{\gamma }P_{1}\sigma_{S}^{2}}\;+\;2\Theta_{2j}\sqrt{\bar{\beta }P_{2}\sigma_{S}^{2}}\;\\ & +\;2\Theta_{1j}\Theta_{2j}\sqrt{\bar{\gamma }\bar{\beta }P_{1}P_{2}} \end{array}\right)}. \end{array}$$

The achievable distortion averaged over a time interval {1,2,…,n} is

$$\begin{array}{c} \frac{1}{n}\sum_{j=1}^{n}\frac{\sigma_{S}^{2}\left(\sigma_{Z}^{2}\;+\;\gamma \Theta_{1j}^{2}P_{1}\;+\;\beta \Theta_{2j}^{2}P_{2}\right)}{\left(\begin{array}{cc} & \Theta_{1j}^{2}P_{1}\;+\;\Theta_{2j}^{2}P_{2}\;+\;\sigma_{S}^{2}\;+\;\sigma_{Z}^{2}\;\\ & +\;2\Theta_{1j}\sqrt{\bar{\gamma }P_{1}\sigma_{S}^{2}}\;+\;2\Theta_{2j}\sqrt{\bar{\beta }P_{2}\sigma_{S}^{2}}\;\\ & +\;2\Theta_{1j}\Theta_{2j}\sqrt{\bar{\gamma }\bar{\beta }P_{1}P_{2}} \end{array}\right)}, \end{array}$$

which converges as n→∞ to

(24) $$\begin{array}{c} E\left[\frac{\sigma_{S}^{2}\left(\sigma_{Z}^{2}\;+\;\gamma \Theta_{1}^{2}P_{1}\;+\;\beta \Theta_{2}^{2}P_{2}\right)}{\left(\begin{array}{cc} & \Theta_{1}^{2}P_{1}\;+\;\Theta_{2}^{2}P_{2}\;+\;\sigma_{S}^{2}\;+\;\sigma_{Z}^{2}\;\\ & +\;2\Theta_{1}\sqrt{\bar{\gamma }P_{1}\sigma_{S}^{2}}\;+\;2\Theta_{2}\sqrt{\bar{\beta }P_{2}\sigma_{S}^{2}}\;\\ & +\;2\Theta_{1}\Theta_{2}\sqrt{\bar{\gamma }\bar{\beta }P_{1}P_{2}} \end{array}\right)}\right] \end{array}$$

due to the stationarity and ergodicity of the fading processes. This concludes the proof of achievability.

## 5. Converse

In this section, we prove that any successful scheme (that has a vanishing probability of error and is within the distortion tolerance) must satisfy the rate-distortion constraints of Theorem 1. This is proved in two subsections: in Section 5.1, we construct an outer bound on the rate-distortion trade-off region. Subsequently, we shall prove in the next Section 5.2 that this outer bound is achievable, thereby proving Theorem 1.

### 5.1. Outer Bound

The proof of our outer bound is aided by the following lemma, adapted from (Equation (2), [20]).

**Lemma 1.**  *Any communication estimation scheme achieving a distortion $D_{n}≜\frac{1}{n}E\left|\right|S^{n}-{\hat{S}}^{n}{\left|\right|}^{2}$ over blocklength n satisfies*

(25) $$\frac{n}{2}\log\left(\frac{\sigma_{S}^{2}}{D_{n}}\right)\leq I\left(S^{n};Y^{n},\Theta_{1}^{n},\Theta_{2}^{n}\right).$$

**Proof.**  The proof is given in Appendix A.1. □

Another useful property is the differential entropy maximizing property of the Gaussian distribution [40], i.e., for $X_{g}^{n}∼N\left(0,K\right)$,

(26) $$\begin{array}{c} h\left(X^{n}\right)\leq h\left(X_{g}^{n}\right)\;\mathrm{whenever}\;\mathrm{Cov}\left(X^{n}\right)⪯K. \end{array}$$

The above facts will be extensively used in our proofs.

For $\left(\eta_{1},\eta_{2},\lambda \right)\in R_{+}^{3}$, we define

$$L\left(\eta_{1},\eta_{2},\lambda \right)=\max\eta_{1}R_{1}+\eta_{2}R_{2}+\frac{\lambda }{2}\log\frac{\sigma_{S}^{2}}{D},$$

where the maximum is over all $\left(R_{1},R_{2},D\right)$ obeying (5)–(8). We note that it suffices to restrict attention to $\eta_{i}\geq 0$, since $\eta_{i}<0$ will trivially correspond to $R_{i}=0$ in the maximization, a case already accounted for by $\eta_{i}=0$. Likewise, since $D\leq \sigma_{S}^{2}$, only λ≥0 need be considered. Therefore, we only consider non-negative weighting coefficients in the sequel. The converse is established by showing that if $\left(R_{1},R_{2},D_{n}\right)$ is achievable using block length *n*, then, for all $\eta_{1},\eta_{2},\lambda \geq 0$,

(27) $$\begin{array}{c} \eta_{1}R_{1}+\eta_{2}R_{2}+\frac{\lambda }{2}\log\frac{\sigma_{S}^{2}}{D_{n}}\leq L\left(\eta_{1},\eta_{2},\lambda \right)+o\left(1\right), \end{array}$$

where o(1) has the usual meaning in standard Landau notation. We note that since the tuple $\left(W_{1},W_{2},S^{n},\Theta_{1}^{n},\Theta_{2}^{n}\right)$ is independent, the Markov chain $X_{1}^{n}\to S^{n}\to X_{2}^{n}$ holds. Denoting

$$\begin{array}{cc} \sigma_{X|Y^{n}}^{2} & ≜\min_{\alpha \in R^{n\times 1}}E{\left[X-\alpha^{T}Y^{n}\right]}^{2}, \end{array}$$

we have for the *i*-th entry in a block of transmissions,

$$\begin{array}{cc} \sigma_{X_{1i}+X_{2i}|S^{n}}^{2} & =\sigma_{X_{1i}|S^{n}}^{2}+\sigma_{X_{2i}|S^{n}}^{2}. \end{array}$$

We define the *empirical* covariance matrix $K_{i}$ of the vector $\left(X_{1i},X_{2i},S_{i}\right)$ with $K_{i}\left(p,l\right)$ denoting its entries. Let us denote

$$\begin{array}{cc} K_{i}\left(j,j\right) & =E\left[X_{ji}^{2}\right]=P_{ji},j=1,2 \end{array}$$

where $P_{ji},j=1,2$ satisfy the power constraints

$$\begin{array}{c} P_{1}\geq \frac{1}{n}\sum_{i=1}^{n}P_{1i},\\ P_{2}\geq \frac{1}{n}\sum_{i=1}^{n}P_{2i}. \end{array}$$

Next, we introduce two parameters $\gamma_{i}\in \left[0,1\right]$ and $\beta_{i}\in \left[0,1\right]$ such that

(28) $$\begin{array}{c} \sigma_{X_{1i}|S^{n}}^{2}≜\gamma_{i}P_{1i}, \end{array}$$

(29) $$\begin{array}{c} \sigma_{X_{2i}|S^{n}}^{2}≜\beta_{i}P_{2i}. \end{array}$$

We now define two parameters γ∈[0,1] and β∈[0,1] such that

(30) $$\begin{array}{cc} \gamma & =\frac{1}{nP_{1}}\sum_{i=1}^{n}\gamma_{i}P_{1i},\\ \beta & =\frac{1}{nP_{2}}\sum_{i=1}^{n}\beta_{i}P_{2i}. \end{array}$$

With this, we are ready to prove (27). Firstly, considering the case where $\eta_{1}\geq \eta_{2}$ is sufficient, as a simple renaming of the indices will give us the other case. For $\eta_{2}>0$, since λ is an arbitrary positive number, maximizing the left-hand side of (27) is equivalent to maximizing $\eta_{1}R_{1}+\eta_{2}R_{2}+\eta_{2}\lambda \frac{1}{2}\log\frac{\sigma_{S}^{2}}{D_{n}}$. Dividing by $\eta_{2}$, and then renaming $\frac{\eta_{1}}{\eta_{2}}$ as η, the maximization becomes ∀η≥1,λ≥0,

(31) $$\begin{array}{c} \max\;\eta R_{1}+R_{2}+\frac{\lambda }{2}\log\frac{\sigma_{S}^{2}}{D_{n}}. \end{array}$$

**Figure 3:**
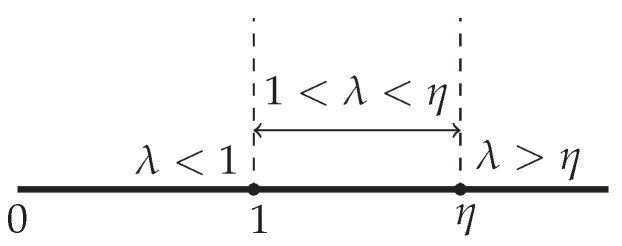
Range of λ for a given η.

For a given η>1, three regimes of λ arise, as shown in Figure 3. Let $R_{1}\left(\gamma \right),R_{2}\left(\beta \right),R_{sum}\left(\gamma ,\beta \right)$ and D(γ,β), respectively, denote the right-hand side of Equations (5)–(8). The following two lemmas are crucial to our proofs.

**Lemma 2.**  *For λ≤1, and γ,β defined in (30), we have*

(32) ηR1+R2+λ2logσS2Dnw≤(η−1)R1(γ)+Rsum(γ,β)+λ2logσS2D(γ,β)+o(1).

**Proof.**  The proof is given in Appendix A.2. □

**Lemma 3.**  *The function $g\left(\gamma ,\beta \right):=R_{sum}\left(\gamma ,\beta \right)+\frac{1}{2}\log\frac{\sigma_{S}^{2}}{D(\gamma ,\beta )}$ is a non-increasing function in each of the arguments when the other argument is held fixed, for γ∈[0,1] and β∈[0,1].*

**Proof.**  We first note that D(γ,β) increases with γ (or β), see (8). Furthermore, a straightforward inspection reveals that g(γ,β) is non-increasing in each of the arguments. □

We now consider the different regimes for λ (see Figure 3).

**Case 1**  (λ≤1andη≥1): In this regime, Lemma 2 directly gives a bound on the weighted sum rate.
**Case 2**  (λ≥ηandη≥1): Since η≥1, we have
  (33) $$\begin{array}{cc} \; & \eta R_{1}+R_{2}+\frac{\lambda }{2}\log\frac{\sigma_{S}^{2}}{D_{n}}\leq \eta R_{1}+\eta R_{2}+\frac{\lambda }{2}\log\frac{\sigma_{S}^{2}}{D_{n}}\\ \; & =\eta \left(R_{1}+R_{2}\right)+\frac{\lambda }{2}\log\frac{\sigma_{S}^{2}}{D_{n}}\\ \; & =\eta \left(R_{1}+R_{2}+\frac{1}{2}\log\frac{\sigma_{S}^{2}}{D_{n}}\right)+\frac{\lambda -\eta }{2}\log\frac{\sigma_{S}^{2}}{D_{n}}\\ \; & \overset{\left(a\right)}{\leq }\eta \left(R_{sum}\left(0,0\right)+\frac{1}{2}\log\frac{\sigma_{S}^{2}}{D(0,0)}\right)+\frac{\lambda -\eta }{2}\log\frac{\sigma_{S}^{2}}{D_{n}}\\ \; & \overset{\left(b\right)}{\leq }0+\frac{\eta }{2}\log\frac{\sigma_{S}^{2}}{D(0,0)}+\frac{\lambda -\eta }{2}\log\frac{\sigma_{S}^{2}}{D(0,0)}, \end{array}$$
  where (a) follows from an application of Lemma 2 followed by Lemma 3, and (b) follows from the fact that uncoded transmission of the state by the two users acting as a super-user with power ${\left(\sqrt{P_{1}}+\sqrt{P_{2}}\right)}^{2}$ results in the minimal distortion possible [20].
**Case 3**  (1≤λ≤ηandη≥1): Since λ≥1, we have

Since λ≥1, we have

$$\begin{array}{cc} \; & \eta R_{1}+R_{2}+\frac{\lambda }{2}\log\frac{\sigma_{S}^{2}}{D_{n}}\leq \eta R_{1}+\lambda R_{2}+\frac{\lambda }{2}\log\frac{\sigma_{S}^{2}}{D_{n}}\\ \; & =\left(\eta -\lambda \right)R_{1}+\lambda \left(R_{1}+R_{2}+\frac{1}{2}\log\frac{\sigma_{S}^{2}}{D_{n}}\right)\\ \; & \leq \left(\eta -\lambda \right)R_{1}+\lambda \left(R_{sum}\left(\gamma ,0\right)+\frac{1}{2}\log\frac{\sigma_{S}^{2}}{D(\gamma ,0)}\right), \end{array}$$

where the last step follows from Lemmas 2 and 3. From (A11) in Appendix A.2, it follows that the inequality $R_{1}\leq E_{\Theta_{1}}\left[\frac{1}{2}\log\left(1+\gamma \Theta_{1}^{2}P_{1}/\sigma_{Z}^{2}\right)\right]$ holds. Thus,

(34) $$\begin{array}{cc} \eta R_{1} & +R_{2}+\frac{\lambda }{2}\log\frac{\sigma_{S}^{2}}{D_{n}}\leq \eta R_{sum}\left(\gamma ,0\right)+\frac{\lambda }{2}\log\frac{\sigma_{S}^{2}}{D(\gamma ,0)}. \end{array}$$

We next prove that (32)–(34) define the region in Theorem 1.

### 5.2. Equivalence of Inner and Outer Bounds

We now show that the regions defined by the inner and outer bounds in Section 4 and Section 5.1 coincide, thereby establishing the capacity region. We will consider three regimes for λ≥0 for each η≥1, and prove that the maximal value of $\eta R_{1}+R_{2}+\frac{\lambda }{2}\log\frac{\sigma_{S}^{2}}{D}$ in the outer bound specified by (32)–(34) can be achieved.

While maximizing $\eta R_{1}+R_{2}+\frac{\lambda }{2}\log\frac{\sigma_{S}^{2}}{D_{n}}$, we already proved that λ≥η corresponds to an extreme point with zero sum-rate (Case 2 in Section 5.1). Clearly, the corresponding distortion lower bound D(0,0) for this case specified by (33) can be achieved by uncoded state transmission by both transmitters using all the available powers. As a result, the condition λ=η encompasses all λ≥η. Moreover, the regime 1≤λ≤η (Case 3 of Section 5.1) corresponds to the case where $R_{2}=0$. This implies that we only need to consider λ=1 rather than λ∈[1,η). Clearly, the region with $R_{2}=0$ follows from the single-user results of [35], but for a state process with variance ${\left(\sqrt{P_{2}}+\sqrt{\sigma_{S}^{2}}\right)}^{2}$. This proves the achievability of the bound specified by (34). This leaves us with proving the achievability for those cases in which 0<λ<1 holds, corresponding to (32). In this regime, the following lemma is crucial.

**Lemma 4.**  *For (0<λ<1,η≥1), the function $k\left(\gamma ,\beta \right):=\left(\eta -1\right)R_{1}\left(\gamma \right)+R_{sum}\left(\gamma ,\beta \right)+\frac{\lambda }{2}\log\frac{\sigma_{S}^{2}}{D(\gamma ,\beta )}$ is jointly strictly concave in (γ,β) for 0≤γ≤1 and 0≤β≤1.*

**Proof.**  The proof is given in Appendix A.3. □

Since we know that $\eta R_{1}+R_{2}+\frac{\lambda }{2}\log\frac{\sigma_{S}^{2}}{D_{n}}\leq k\left(\gamma ,\beta \right)$ for some value of (γ,β)∈[0,1]×[0,1], the strict concavity of k(·) suggests the existence of a unique $\left(\gamma^{*},\beta^{*}\right)$ for which $\eta R_{1}+R_{2}+\frac{\lambda }{2}\log\frac{\sigma_{S}^{2}}{D_{n}}$ is maximized for the given η>1 and 0≤λ≤1. Evidently, choosing these maximizing parameters $\left(\gamma^{*},\beta^{*}\right)$ in our achievable theorem will give us the same operating point. Reversing the roles of $R_{1}$ and $R_{2}$, we have covered the whole region. Thus, we have established the achievability of the outer bound specified with (32)–(34). This completes the proof of Theorem 1.

## 6. Conclusions

We investigated joint message transmission and state estimation in a state-dependent fading Gaussian multiple access channel in this paper and characterized the trade-off region between message rates and state estimation distortion. It was shown that the optimal strategy involved static power allocation and uncoded state amplification combined with Gaussian signaling and dirty paper coding. While the role of uncoded communications has been examined before for non-fading settings without state dependence, ours is the first result that brings out its significance in the context of state-dependent fading systems.

Our framework naturally generalizes previous results concerning state estimation on point-to-point fading channels to multiple users as well as point-to-point non-fading settings to fading links with multiple users. Our results contribute to a better understanding of joint state estimation and communication problems in multi-terminal settings. They can be used as design guidelines for practical systems employing joint sensing and communication envisioned in future 6G wireless standards and broadly applies to systems that involve joint compression and communication/rate-distortion trade-offs.

However, we assumed perfect state observation at the transmitters in this work. A long-standing open problem is that of communicating state and message streams in a fading GMAC with noisy state observations at the transmitters, which is left for future work. Moreover, there could be settings when the receiver cannot track the channel fading gains either, unlike this work. Thus, another interesting avenue for further research is an investigation of the current setup when the encoders, as well as the decoder, are totally uninformed about the fading coefficients on the links.

## Figures and Tables

**Table 1:** Summary of paper contributions. Note that single-user (noncausal) refers to a point-to-point state-dependent channel with noncausal transmitter state information, BC (causal) refers to a state-dependent broadcast channel with causal transmitter state information, while MAC (noncausal) refers to a state-dependent multiple access channel with noncausal state information at all the transmitters.

	Single-User (Noncausal)		BC (Causal)	MAC (Noncausal)	
	**No Fading**	**Fading**	**No Fading**	**No Fading**	**Fading**
No State Estimation	[17]	[32]	[36]	[37]	This work
State Estimation	[20]	This work	[28]	[25]	This work

## Data Availability

Not applicable.

## Appendix A

### Appendix A.1. Proof of Lemma 1

The proof is along the lines of (Equation (2), [20]), with appropriate modifications to incorporate the fading coefficients $\left(\Theta_{1}^{n},\Theta_{2}^{n}\right)$. We provide a full proof for completeness. Consider the following chain of inequalities, starting with the right-hand side in Lemma 1:

(A1) $$\begin{array}{cc} \; & \frac{1}{n}I\left(S^{n};Y^{n},\Theta_{1}^{n},\Theta_{2}^{n}\right)\\ \; & =\frac{1}{n}\left(h\left(S^{n}\right)-h\left(S^{n}|Y^{n},\Theta_{1}^{n},\Theta_{2}^{n}\right)\right)\\ \; & \overset{\left(a\right)}{=}\frac{1}{n}\left(h\left(S^{n}\right)-h\left(S^{n}-{\hat{S}}^{n}\left(Y^{n},\Theta_{1}^{n},\Theta_{2}^{n}\right)|Y^{n},\Theta_{1}^{n},\Theta_{2}^{n}\right)\right)\\ \; & \geq \frac{1}{n}\left(h\left(S^{n}\right)-h\left(S^{n}-{\hat{S}}^{n}\right)\right)\\ \; & \overset{\left(b\right)}{=}\frac{1}{n}\left(\sum_{i=1}^{n}h\left(S_{i}\right)-h\left(S^{n}-{\hat{S}}^{n}\right)\right)\\ \; & \geq \frac{1}{n}\sum_{i=1}^{n}\left(h\left(S_{i}\right)-h\left(S_{i}-{\hat{S}}_{i}\right)\right)\\ \; & =\frac{1}{n}\sum_{i=1}^{n}\left(\frac{1}{2}\log\left(2\pi e\sigma_{S}^{2}\right)-h\left(S_{i}-{\hat{S}}_{i}\right)\right)\\ \; & \overset{\left(c\right)}{\geq }\frac{1}{n}\sum_{i=1}^{n}\left(\frac{1}{2}\log\left(2\pi e\sigma_{S}^{2}\right)-\frac{1}{2}\log\left(2\pi eE{\left(S_{i}-{\hat{S}}_{i}\right)}^{2}\right)\right)\\ \; & \overset{\left(d\right)}{\geq }\frac{1}{2}\log\left(2\pi e\sigma_{S}^{2}\right)-\frac{1}{2}\log\left(2\pi e\frac{1}{n}\sum_{i=1}^{n}E{\left(S_{i}-{\hat{S}}_{i}\right)}^{2}\right)\\ \; & =\frac{1}{2}\log\left(2\pi e\sigma_{S}^{2}\right)-\frac{1}{2}\log\left(2\pi e\frac{1}{n}E{∥S^{n}-{\hat{S}}^{n}∥}^{2}\right)\\ \; & =\frac{1}{2}\log\left(\frac{\sigma_{S}^{2}}{\frac{1}{n}E{∥S^{n}-{\hat{S}}^{n}∥}^{2}}\right)\\ \; & =\frac{1}{2}\log\left(\frac{\sigma_{S}^{2}}{D_{n}}\right), \end{array}$$

where (a) follows since ${\hat{S}}^{n}\left(Y^{n},\Theta_{1}^{n},\Theta_{2}^{n}\right)$ is a function of $\left(Y^{n},\Theta_{1}^{n},\Theta_{2}^{n}\right)$, (b) follows since $S^{n}$ is i.i.d., (c) follows since the Gaussian distribution maximizes differential entropy for a given variance, while (d) follows from Jensen’s inequality.

### Appendix A.2. Proof of Lemma 2

By Fano’s inequality [40], we can write for any ϵ>0, for large enough *n*, $H(W_{1},W_{2}|Y^{n})\leq n\epsilon$. Then, we have

(A2) nηR1+nR2+nλ2logσS2Dn=n(η−1)R1+n∑j=12Rj+nλ2logσS2Dn≤(a)(η−1)H(W1)+H(W1,W2)+λI(Sn;Yn,Θ1n,Θ2n)=(b)(η−1)H(W1|X2n,Sn,Θ1n)+H(W1,W2|Sn,Θ1n,Θ2n)www+λI(Sn;Yn,Θ1n,Θ2n)=(c)(η−1)H(W1|X2n,Sn,Θ1n)+H(W1,W2|Sn,Θ1n,Θ2n)www+λI(Sn;Yn|Θ1n,Θ2n)≤(d)(η−1)I(W1;Yn|X2n,Sn,Θ1n)+nϵww+I(W1,W2;Yn|Sn,Θ1n,Θ2n)+nϵ+λI(Sn;Yn|Θ1n,Θ2n)=(η−1)I(W1;Yn|X2n,Sn,Θ1n)www+λI(W1,W2,Sn;Yn|Θ1n,Θ2n)www+(1−λ)I(W1,W2;Yn|Sn,Θ1n,Θ2n)+2nϵ=(η−1)(h(Yn|X2n,Sn,Θ1n)−h(Yn|W1,X2n,Sn,Θ1n))w+λ(h(Yn|Θ1n,Θ2n)−h(Yn|W1,W2,Sn,Θ1n,Θ2n))w+(1−λ)(h(Yn|Sn,Θ1n,Θ2n)wwwwwwww−h(Yn|W1,W2,Sn,Θ1n,Θ2n))+2nϵ≤∑i=1n(η−1)(h(Yi|X2i,Si,Θ1i)−h(Zi))w+∑i=1n{λh(Yi|Θ1i,Θ2i)+(1−λ)h(Yi|Si,Θ1i,Θ2i)wwwwwwwwwwwwwwwwwwwwwww−h(Zi)}+2nϵ=E∑i=1n(η−1)(h(Yi|X2i,Si,Θ1i=Θ1)−h(Zi))w+E[∑i=1n(λh(Yi|Θ1i=Θ1,Θ2i=Θ2)wwwww+(1−λ)h(Yi|Si,Θ1i=Θ1,Θ2i=Θ2)wwwwwwwwwwwwwwwwwwwwwww−h(Zi))]+2nϵ,

where (a) uses Lemma 1 and the fact that the messages are uniformly distributed on their respective alphabets, (b) follows since $\left(W_{1},W_{2}\right)⫫\left(S^{n},\Theta_{1}^{n},\Theta_{2}^{n}\right)$ and $W_{1}⫫\left(X_{2}^{n},S^{n},\Theta_{1}^{n}\right)$, (c) follows since $\left(\Theta_{1}^{n},\Theta_{2}^{n}\right)⫫S^{n}$ and (d) follows from Fano’s inequality. We now upper bound the term $\lambda h\left(Y_{i}|\Theta_{1i}=\Theta_{1},\Theta_{2i}=\Theta_{2}\right)+\left(1-\lambda \right)h\left(Y_{i}|S_{i},\Theta_{1i}=\Theta_{1},\Theta_{2i}=\Theta_{2}\right)$ in (A2). Notice that for a given covariance matrix $K_{i}$, $\lambda h\left(Y_{i}|\Theta_{1i}=\Theta_{1},\Theta_{2i}=\Theta_{2}\right)+\left(1-\lambda \right)h\left(Y_{i}|S_{i},\Theta_{1i}=\Theta_{1},\Theta_{2i}=\Theta_{2}\right),\;\;\lambda \in \left[0,1\right]$, is simultaneously maximized when $\left(\Theta_{1}X_{1i}+\Theta_{2}X_{2i}\right)$ is jointly Gaussian with $S_{i}$.

Without loss of generality, for the purposes of finding an upper bound on $\eta R_{1}+R_{2}+\frac{\lambda }{2}\log\frac{\sigma_{S}^{2}}{D_{n}}$, we can express $X_{ji}$ in terms of its linear least squares estimate given $S^{n}$ and an error term. Therefore,

(A3) $$\begin{array}{c} X_{ji}=V_{ji}+W_{ji}, \end{array}$$

where $V_{ji}$ is uncorrelated with $S^{n}$, and $W_{ji}=\sum_{k=1}^{n}\beta_{jik}S_{k}$ for appropriate coefficients $\left\{\beta_{jik},k\in \left\{1,2,\ldots ,n\right\}\right\}$. But it is readily verified from expressions (28), (29) and (A3) that $E\left[W_{1i}^{2}\right]=\left(1-\gamma_{i}\right)P_{1i}$ and $E\left[W_{2i}^{2}\right]=\left(1-\beta_{i}\right)P_{2i}$. Now, for a fixed $E\left[W_{1i}^{2}\right]=\left(1-\gamma_{i}\right)P_{1i}$, it follows that the variance of $\Theta_{1}X_{1i}+S_{i}=\Theta_{1}V_{1i}+\left(\Theta_{1}W_{1i}+S_{i}\right)$ would be maximized when $\Theta_{1}W_{1i}$ is a scaled version of $S_{i}$, i.e.,

(A4) $$\begin{array}{c} \Theta_{1}X_{1i}=\Theta_{1}V_{1i}\;+\;\mu_{1i}\Theta_{1}\sqrt{\left(1\;-\;\gamma_{i}\right)\frac{P_{1i}}{\sigma_{S}^{2}}}S_{i}, \end{array}$$

where $\mu_{1i}\in \left\{-1,+1\right\}$ is the sign of the correlation between $X_{1i}$ and $S_{i}$. Likewise, we can express

(A5) $$\begin{array}{c} \Theta_{2}X_{2i}=\Theta_{2}V_{2i}\;+\;\mu_{2i}\Theta_{2}\sqrt{\left(1\;-\;\beta_{i}\right)\frac{P_{2i}}{\sigma_{S}^{2}}}S_{i}, \end{array}$$

where $\mu_{2i}\in \left\{-1,+1\right\}$ is the sign of the correlation between $X_{2i}$ and $S_{i}$. Adding expressions (A4) and (A5), we obtain

(A6) ∑j=12ΘjXji=Vi+μ1iΘ1(1−γi)P1iσS2wwwwww+μ2iΘ2(1−βi)P2iσS2Si,

where $V_{i}=\Theta_{1}V_{1i}+\Theta_{2}V_{2i}$ is zero mean Gaussian, and independent of $S^{n}$. The second term on the right-hand side of (A6) can be understood as the linear estimate of $\left(\Theta_{1}X_{1i}+\Theta_{2}X_{2i}\right)$ given $S_{i}$. Since $V_{i}=\Theta_{1}V_{1i}+\Theta_{2}V_{2i}$ and $S^{n}$ are independent, it follows from (28) and (29) that

$$\begin{array}{cc} \mathrm{Var}[V_{i}] & =\sigma_{\Theta_{1}X_{1i}+\Theta_{2}X_{2i}|S^{n}}^{2}\\ \; & =\gamma_{i}\Theta_{1}^{2}P_{1i}+\beta_{i}\Theta_{2}^{2}P_{2i}. \end{array}$$

Using this, it follows that

$$\begin{array}{cc} E\left[{\left(\Theta_{1}X_{1i}+\Theta_{2}X_{2i}\right)}^{2}\right] & \leq \Theta_{1}^{2}P_{1i}+\Theta_{2}^{2}P_{2i}\\ \; & \;\;\;\;\;\;\;\;\;\;\;\;\;\;\;\;\;\;+2\Theta_{1}\Theta_{2}\sqrt{\left(1-\gamma_{i}\right)\left(1-\beta_{i}\right)P_{1i}P_{2i}}, \end{array}$$

where we have taken $\mu_{1i}=\mu_{2i}=1$ as the sign of correlation in (A6), since negative correlation can only be detrimental for the right-hand side. Denoting κ(x)=(1/2)log(2πex) and using the differential entropy maximizing property of Gaussian random variables for a given variance, we have

(A7) $$\begin{array}{cc} h(Y_{i}|X_{2i},S_{i},\Theta_{1i}=\Theta_{1}) & \leq \kappa (\gamma_{i}\Theta_{1}^{2}P_{1i}+\sigma_{Z}^{2}), \end{array}$$

(A8) h(Yi|Si,Θ1i=Θ1,Θ2i=Θ2)wwww≤κ(γiΘ12P1i+βiΘ22P2i+σZ2),

(A9) h(Yi|Θ1i=Θ1,Θ2i=Θ2)www≤κ(Θ12P1i+Θ22P2i+σS2+σZ2wwwwwww+2Θ1γ¯iP1iσS2+2Θ2β¯iP2iσS2wwwwwww+2Θ1Θ2γ¯iβ¯iP1iP2i),

where (A8) is under the choice of $\left(\Theta_{1}X_{1i}+\Theta_{2}X_{2i}\right)$ which maximizes $\lambda h\left(Y_{i}|\Theta_{1i}=\Theta_{1},\Theta_{2i}=\Theta_{2}\right)+\left(1-\lambda \right)h\left(Y_{i}|S_{i},\Theta_{1i}=\Theta_{1},\Theta_{2i}=\Theta_{2}\right)$, for all λ∈[0,1]. Continuing the sequence of inequalities from (A2):

(A10) nηR1+nR2+nλ2logσS2Dn−2nϵ≤(a)E∑i=1n(η−1)12logγiΘ12P1i+σZ2σZ2−n2log(σZ2)w+E∑i=1nλ2logΘ12P1i+Θ22P2i+σS2+σZ2+2Θ1γ¯iP1iσS2+2Θ2β¯iP2iσS2+2Θ1Θ2γ¯iβ¯iP1iP2iw+E∑i=1n(1−λ)2logγiΘ12P1i+βiΘ22P2i+σZ2≤(b)E(η−1)n2logγΘ12P1+σZ2σZ2−n2log(σZ2)w+Eλn2logΘ12P1+Θ22P2+σS2+σZ2+2Θ1γ¯P1σS2+2Θ2β¯P2σS2+2Θ1Θ2γ¯β¯P1P2w+En(1−λ)2logγΘ12P1+βΘ22P2+σZ2=(c)E(η−1)n2logγΘ12P1+σZ2σZ2ww+nRsum(γ,β)+λn2logσS2D(γ,β).

where (a) follows from the fact that both λ and (1−λ) are non-negative for λ∈[0,1] and expressions (A7)–(A9), (b) follows from Jensen’s Inequality, and (c) follows from the definitions of $R_{sum}\left(\gamma ,\beta \right)$, D(γ,β) from (7) and (8). Thus, the lemma is proved for all λ∈[0,1]. Following similar lines, the individual rate $R_{1}$ can be bounded using Fano’s inequality and (A7), to obtain

(A11) $$\begin{array}{c} R_{1}\leq E\left[\frac{1}{2}\log\left(\frac{\gamma \Theta_{1}^{2}P_{1}+\sigma_{Z}^{2}}{\sigma_{Z}^{2}}\right)\right]. \end{array}$$

### Appendix A.3. Proof of Lemma 4

Consider a concave function $T(\underline{\omega })$, where $\underline{\omega }=\left[\omega_{1}\;\omega_{2}\ldots \omega_{N}\right]\in {\left[0,1\right]}^{N}$, and define

(A12) $$\begin{array}{c} f\left(\underline{\omega }\right):=\sum_{i=1}^{N}\delta_{i}\log\left(1+\sum_{j=1}^{i}\omega_{j}P_{j}\right)+\frac{\lambda }{2}\log T\left(\underline{\omega }\right), \end{array}$$

where $\delta_{i},1\leq i\leq N$ and λ are non-negative constants.

**Lemma A1.** 
*For 0<λ≤1, f(·) is strictly concave in $\underline{\omega }\in {\left[0,1\right]}^{N}$, whenever $\delta_{i},1\leq i\leq N$ are not identically zero.*

**Proof.** The first term, which is a linear combination of logarithms, is strictly concave. We next consider the second term. Let $\underline{y_{1}}$ and $\underline{y_{2}}$ be two N− dimensional vectors in $R^{N}$. Notice that for ι∈[0,1],

(A13) ιlogT(y1_)+(1−ι)logT(y2_)wwwwwwwwwwwwwww≤log(ιT(y1_)+(1−ι)T(y2_))wwwwwwwwwwwwwww≤logT(ιy1_+(1−ι)y2_),

since T(·) itself is concave by assumption. □

Let us now proceed to prove Lemma 4. We denote

(A14) T(γ,β)=Θ12P1+Θ22P2+σS2+σZ2w+2Θ1γ¯P1σS2+2Θ2β¯P2σS2w+2Θ1Θ2γ¯β¯P1P2,

for convenience. Note that the function

(A15) k′(γ,β)≜(η−1)2log1+γΘ12P1σZ2www+λ2logT(γ,β)σZ2www+(1−λ)2log1+γΘ12P1+βΘ22P2σZ2

is a sum similar to (A12). We first show that $k^{\prime }\left(\gamma ,\beta \right)$ is strictly concave by proving T(·) in (A14) to be a concave function. For $d_{0}>0$, and non-negative constants $d_{1},\cdots ,d_{N}$, the function

$$T\left(\underline{\omega }\right)=d_{0}+\sum_{i=1}^{N}\sum_{j=1}^{N}d_{i}d_{j}\sqrt{\left(1-\omega_{i}\right)(1-\omega_{j}})$$

is concave. To see this, we note that $\sqrt{x}$ is strictly concave in x≥0. Moreover, $\sqrt{xz}$ is jointly concave in $\left(x,z\right)\in {\left[0,1\right]}^{2}$, implying that T(ω) is a concave function. Notice that concavity in the range of interest is unaffected if every variable x∈[0,1] is replaced by 1−x. Finally, it follows that the function in Lemma 4, $k\left(\gamma ,\beta \right)=E[k^{\prime }\left(\gamma ,\beta \right)]$, is strictly concave as well as a result of Jensen’s inequality. This concludes the proof of the lemma.
